# Excessively High Maternal Total Cell-Free DNA Concentration and Fetal Growth Restriction in Singleton Pregnancies: A Retrospective Cohort Study

**DOI:** 10.3390/jcm15176798

**Published:** 2026-09-02

**Authors:** Lingling Xing, Xiaosha Jing, Ting Bai, Sha Liu, Jianlong Liu, Cechuan Deng, Tianyu Xia, Yunyun Liu, Xiang Wei, Yuan Luo, Quanfang Zhou, Qian Zhu, Hongqian Liu

**Affiliations:** 1 Center for Medical Genetics, West China Second University Hospital, Sichuan University, Chengdu 610041, China; xinglingling@motherchildren.com (L.X.); ss202609@163.com (X.J.); baiting3033@163.com (T.B.); liusha63hxcq@163.com (S.L.); 1988liujianlong@163.com (J.L.); dengcechuan@163.com (C.D.); xing_002026@163.com (T.X.); liuyunyun2010@163.com (Y.L.); ljweixiang@aliyun.com (X.W.); 15208225836@163.com (Y.L.); wuqilongfang576@icloud.com (Q.Z.); 2 Key Laboratory of Birth Defects and Related Diseases of Women and Children (Sichuan University), Ministry of Education, Chengdu 610041, China

**Keywords:** maternal total cell-free DNA, fetal growth restriction, fetal weight, propensity score matching

## Abstract

**Background/Objectives:** Noninvasive prenatal screening (NIPS) fails in approximately 0.2% of cases due to high maternal plasma total cell-free DNA (t-cfDNA) concentration (≥0.6 ng/µL) confirmed after two DNA extraction attempts. The clinical implications of this quality control metric for fetal growth restriction (FGR) remain unknown. **Methods:** This retrospective cohort study, conducted in a major prenatal diagnosis center in China, involved pregnant women who underwent noninvasive prenatal screening (NIPS) between 2019 and 2024. From a total of 73,636 pregnancies, 102 singleton pregnancies with excessively high t-cfDNA concentration (≥0.6 ng/µL upon retesting) leading to NIPS failure were compared with 396 matched controls (t-cfDNA < 0.6 ng/µL) after propensity score matching. The primary outcome was FGR, defined as estimated fetal weight or abdominal circumference below the 10th percentile for gestational age. **Results:** In the matched cohort, FGR occurred in 26.47% of the high-concentration group versus 9.60% of controls (OR = 3.27, 95% CI: 1.45–7.41, *p* = 0.004). The high-concentration group also had lower gestational age at delivery (37.44 vs. 38.48 weeks, *p* < 0.001) and higher preterm birth rates (18.63% vs. 7.57%, *p* = 0.025). Subgroup analyses for severe and FGR first detected before 32 weeks were exploratory and limited by statistical power, warranting cautious interpretation. **Conclusions**: An excessively high maternal t-cfDNA concentration in early pregnancy shows a significant association with FGR and adverse pregnancy outcomes. These findings suggest that t-cfDNA could potentially serve as an early biomarker for identifying pregnancies at elevated risk of FGR. Further validation of our findings in prospective studies is required to establish its clinical utility.

## 1. Introduction

Fetal growth restriction (FGR), defined by the American College of Obstetricians and Gynecologists (ACOG) as an estimated fetal weight (EFW) or abdominal circumference (AC) below the 10th percentile for gestational age, affects approximately 5–10% of all pregnancies and is a major contributor to perinatal morbidity and mortality [1,2]. Affected infants face increased risks of neurodevelopmental delay, neonatal hypoglycemia, hypothermia, and long-term metabolic and cardiovascular diseases [2,3,4]. A significant challenge in modern obstetrics is the identification of reliable biomarkers for the early prediction of FGR. Current screening methods in early pregnancy, including maternal characteristics and uterine artery Doppler, lack sufficient sensitivity, while ultrasound diagnosis of abnormal growth is typically feasible only in the second trimester or later [5,6].

In 1997, Lo et al. first identified cell-free fetal DNA (cffDNA) fragments within maternal plasma [7]. Noninvasive prenatal screening (NIPS) based on cell-free DNA (cfDNA) analysis has become a standard approach for detecting fetal aneuploidies. A critical quality control metric in NIPS is the fetal fraction (FF)—the percentage of cffDNA relative to total cell-free DNA (t-cfDNA). FF is significantly influenced by maternal t-cfDNA concentration; any condition that increases the maternal contribution and/or decreases the fetal/placental contribution may lead to a reduction in FF [8,9].

In our center, approximately 0.2% of NIPS cases fail due to high maternal plasma t-cfDNA concentration (≥0.6 ng/µL) confirmed after at least two DNA extraction attempts, according to our institutional standard operating procedure (SOP) for NIPS quality control. These women currently receive no genetic information from NIPS, and the clinical implications of this finding remain poorly understood.

The biological rationale for investigating t-cfDNA is supported by evidence that maternal cfDNA serves as a biomarker of systemic inflammation and cellular injury [10,11,12]. Elevated t-cfDNA may therefore signal a pro-inflammatory state that predisposes to placental dysfunction and adverse pregnancy outcomes. Low FF has been associated with an elevated risk of adverse obstetric outcomes, including FGR [13,14,15,16]. However, the clinical utility of FF is limited, as it is calculated after bioinformatic analysis. In contrast, t-cfDNA concentration—the pool from which FF is derived—is quantified as a routine pre-sequencing quality control step, making it available days earlier. Studies directly investigating the association between t-cfDNA and FGR remain limited, and existing studies have yielded conflicting results [11,17]. We therefore hypothesized, based on the above biological evidence, that high maternal t-cfDNA concentration in early pregnancy is not merely a technical reason for NIPS failure but an early biomarker of maternal-placental pathology predisposing to subsequent FGR.

Using a large cohort from West China Second University Hospital, we investigated whether high t-cfDNA (≥0.6 ng/µL) is associated with increased FGR risk, and whether this association is independent of established FGR risk factors and late-pregnancy complications, including preeclampsia, through propensity score matching and sensitivity analyses.

## 2. Materials and Methods

### 2.1. Laboratory Protocol

Maternal peripheral blood (8–10 mL) was collected in Cell-free DNA BCT tubes (Streck, Omaha, NE, USA). Samples were allowed to stand at room temperature (<25 °C) for 30 min and then stored at 4 °C. Within 72 h of collection, blood samples were centrifuged at 1600× *g* for 10 min at 4 °C. The plasma fraction was transferred into 1.5 mL Eppendorf tubes and further centrifuged at 16,000× *g* for 10 min at 4 °C. The resulting cell-free plasma supernatant was then aliquoted into three 1.5 mL Eppendorf tubes and stored at −70 °C until further analysis.

Cell-free DNA was extracted from 1200 µL of plasma using a commercial DNA extraction kit (Berry Genomics, Hangzhou, China), following the manufacturer‘s instructions. Quantification was performed using a Qubit 3.0 fluorometer with the Ex-Kubit dsDNA HS assay kit (Ex-Cell Biotech, Shanghai, China). The t-cfDNA concentration was recorded for each sample. According to our institutional standard operating procedure (SOP), which is based on the official product insert from Berry Genomics and internal statistical quality control data, a concentration ≥ 0.6 ng/µL confirmed after two independent extraction attempts was defined as high t-cfDNA; concentrations between 0.05 and 0.6 ng/µL were considered qualified.

### 2.2. Study Design and Population

We initially screened all singleton pregnant women who underwent NIPS between 2019 and 2024. A total of 73,636 women were identified. Based on maternal plasma t-cfDNA concentration, women were classified into three groups: the high-concentration group (t-cfDNA ≥ 0.6 ng/µL, *n* = 174), the qualified-concentration group (0.05 ≤ t-cfDNA < 0.6 ng/µL, *n* = 73,320), and the low-concentration group (t-cfDNA < 0.05 ng/µL, *n* = 142). The low-concentration group was not included in this study.

**Figure 1:**
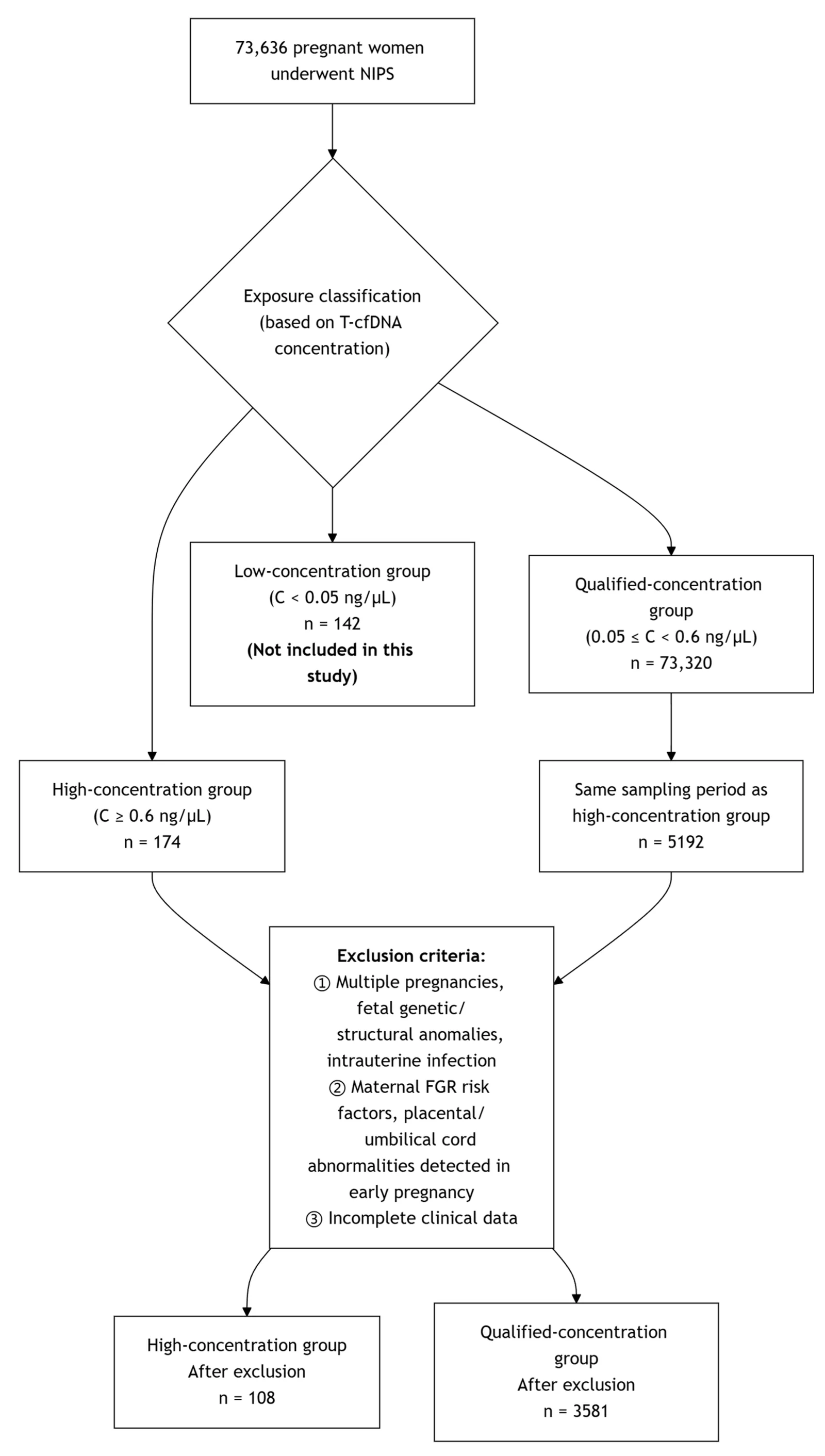
Study flowchart.

From the qualified-concentration group, we selected women who underwent blood sampling during the same period as the high-concentration group to serve as the potential control pool (*n* = 5192), minimizing temporal bias. Women were eligible for inclusion if they had available data on maternal plasma t-cfDNA concentration, completed pregnancy, and documented birth outcomes. To minimize temporal bias, we selected from the qualified-concentration group all women who underwent blood sampling on the same calendar dates as the high-concentration group, yielding a candidate control pool of 5192 women. We then applied the following exclusion criteria simultaneously to both groups before propensity score matching (PSM) to create a baseline population with a relatively homogeneous risk profile, removing individuals with strong, independent risk factors for FGR that could potentially override any signal from t-cfDNA: (1) multiple pregnancies; (2) fetal genetic or structural abnormalities diagnosed either before or after NIPS; (3) severe placental or umbilical cord abnormalities (including severe placental abruption, placenta previa with active bleeding, and true cord knot); (4) confirmed intrauterine infection; and (5) incomplete clinical data (Figure 1).

Following the laboratory protocol described above, women with a t-cfDNA concentration ≥ 0.6 ng/µL confirmed after two DNA extraction attempts were assigned to the high-concentration group. Those with a t-cfDNA concentration between 0.05 and 0.6 ng/µL were assigned to the qualified-concentration group, which served as the control pool.

The primary outcome was FGR, defined according to the ACOG guidelines as estimated fetal weight or abdominal circumference below the 10th percentile for gestational age [1]. Gestational age was determined based on the last menstrual period or the first-trimester ultrasound. Both EFW and AC percentiles were calculated using the fetal growth reference standards developed by the Eunice Kennedy Shriver National Institute of Child Health and Human Development (NICHD), which have been validated for use in Asian populations [18,19]. Severe FGR was defined as EFW or AC below the 3rd percentile for gestational age [20]. FGR first detected before 32 weeks was defined as the first ultrasound detection of FGR (EFW/AC below the 10th percentile per ACOG criteria) occurring before 32 weeks of gestation. All ultrasound examinations were performed by certified obstetric sonographers as part of routine antenatal care. The sonographers were blinded to the NIPS results and to the t-cfDNA concentration status at the time of ultrasound assessment. FGR diagnosis was based on ultrasound measurements at the time of routine visits; no additional protocol-driven ultrasound surveillance was implemented specifically for the high-concentration group.

Secondary outcomes included: (1) gestational age at delivery (weeks); and (2) preterm birth, defined as delivery before 37 completed weeks of gestation. Gestational age at delivery was determined based on the last menstrual period or first-trimester ultrasound.

### 2.3. Statistical Analyses

After applying the exclusion criteria, we performed PSM at a 1:4 ratio (one high-concentration woman matched to four controls) to further balance the remaining baseline covariates between the high- and qualified t-cfDNA groups. The propensity score was estimated using a logistic regression model: Logit(PS) = β_0_ + β_1_ × Age + β_2_ × BMI + β_3_ × Gestational_age + β_4_ × Maternal comorbidities + β_5_ × Fetal factors + β_6_ × Placenta factors. The complete list of variables is provided in Supplementary Table S1.

Matching was performed using the nearest-neighbor method with a caliper of 0.2 standard deviations (SD) of the propensity score. No replacement was allowed.

Before PSM, standard logistic regression was used to estimate the association between high t-cfDNA concentration and FGR. After PSM, we used conditional logistic regression to estimate this association, with FGR as the dependent variable, group (high-concentration vs. qualified-concentration) as the independent variable, and match ID as the stratification variable. Results are presented as odds ratios (ORs) with 95% confidence intervals (CIs).

Subgroup analyses for FGR first detected before 32 weeks and severe FGR were performed using the same regression strategies: binary logistic regression for the unmatched cohort and conditional logistic regression for the matched cohort. Each subtype was compared with women without FGR (EFW/AC ≥ 10th percentile).

To assess the robustness of our primary findings, we conducted sensitivity analyses using multivariable conditional logistic regression that added late-pregnancy maternal and fetal factors (preeclampsia, GDM, and placental abruption) to the primary model. These variables were not included in the primary model as they may lie on the causal pathway. All sensitivity analyses maintained the matched design using conditional logistic regression with the same stratification variable (match ID). Results are presented as adjusted ORs with 95% CIs.

For secondary outcomes, gestational age at delivery was compared between groups using the Mann–Whitney U test, given its non-normal distribution, and the results are presented as medians with interquartile ranges. For preterm birth, conditional logistic regression with match ID as the stratification variable was used to estimate the odds ratio, consistent with the primary analysis. These secondary outcome analyses should be considered descriptive and exploratory.

Data processing and analysis were performed using R version 4.4.1 (R Foundation for Statistical Computing, Vienna, Austria) along with Zstats v1.0 (www.zstats.net) (accessed on 17 August 2026). All statistical tests were two-sided, and *p* < 0.05 was considered statistically significant.

## 3. Results

### 3.1. Study Population and Baseline Characteristics

The proportion of exposed patients lost during 1:4 matching with a caliper of 0.2 SD was 5.56% (6/108). The final analytic cohort comprised 498 women: 102 in the high-concentration group (persistently high t-cfDNA ≥ 0.6 ng/µL) and 396 in the qualified-concentration group (0.05–0.6 ng/µL).

Table 1 presents the baseline characteristics of the study population before and after PSM. Before matching, significant differences between the two groups were observed in maternal age (31.70 ± 3.74 years vs. 30.80 ± 3.24 years, *p* = 0.005) and the prevalence of maternal comorbidities (6.48% vs. 0.67%, *p* < 0.001). Other covariates, including BMI (*p* = 0.172), gestational age at NIPS blood draw (*p* = 0.098), fetal factors (*p* = 0.558), and placental factors (*p* = 1), were comparable between groups.

**Figure 2:**
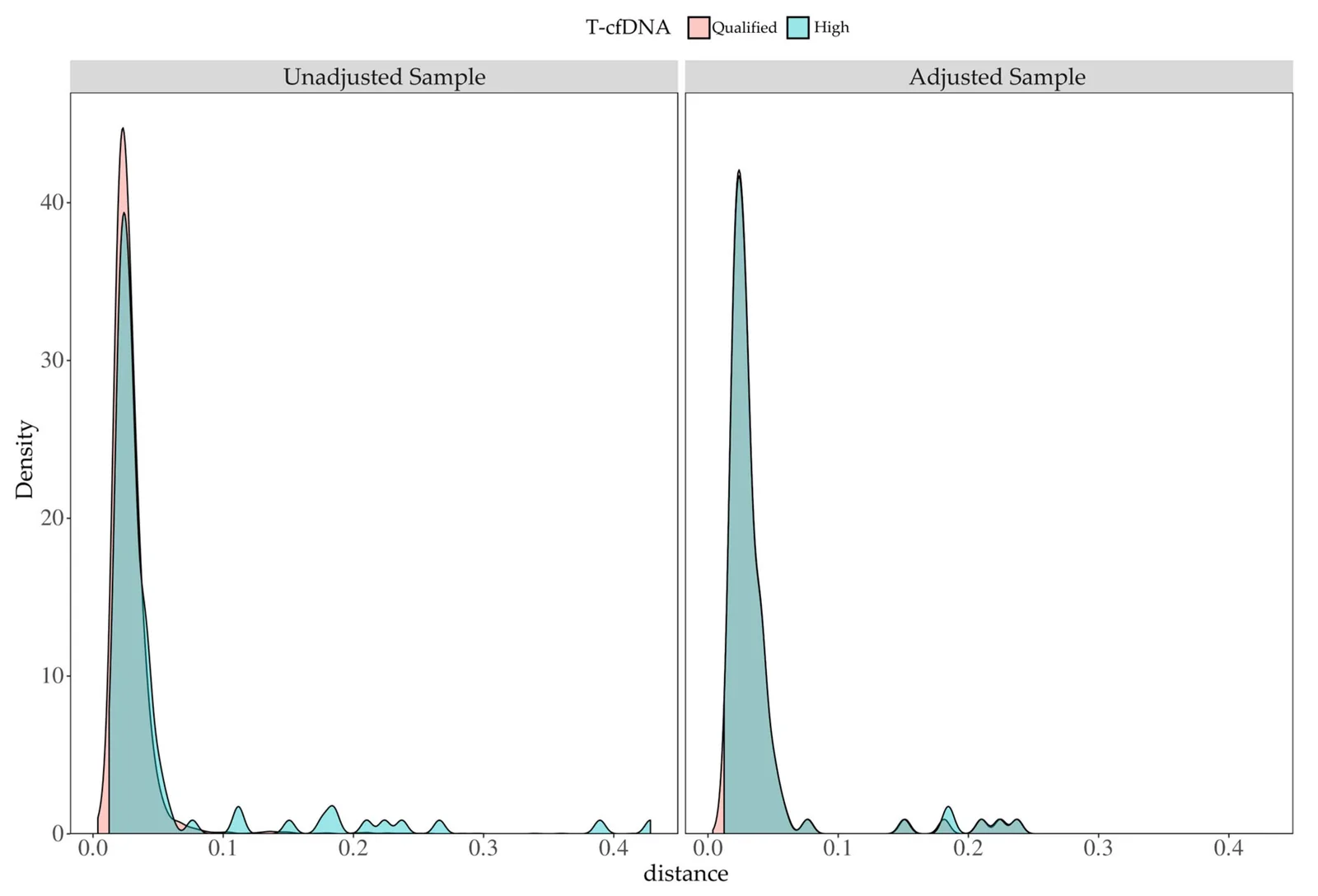
Probability density plots of propensity scores before and after matching.

Figure 2 displays the probability density plots of the propensity scores before and after matching. In the unadjusted sample, the propensity score distribution for the high-concentration group (blue) was shifted to the right compared with that of the qualified-concentration group (pink), indicating that women with high t-cfDNA concentration had systematically higher propensity scores before matching. This imbalance reflected the baseline differences in maternal age and maternal comorbidities observed in Table 1. After 1:4 nearest-neighbor matching with a caliper of 0.2 SD (lower panel), the two propensity score distributions became nearly identical, with substantial overlap and similar central tendencies. The close alignment of the blue and pink curves demonstrates that the matching procedure successfully balanced the pre-exposure covariates between the high-concentration and qualified-concentration groups.

Consistent with the graphical evidence, after matching, all baseline covariates were well balanced between the two groups. As shown in Table 1, no statistically significant differences remained for any variable (all *p* > 0.05), and standardized mean differences (SMDs) were <0.1 for all covariates, indicating adequate balance (Table 1).

### 3.2. Primary Outcome and Sensitivity Analyses

Before matching, the incidence of FGR was 26.85% (29/108) in the high-concentration group versus 9.16% (328/3581) in the control group (unadjusted OR = 3.64, 95% CI: 2.34–5.66, *p* < 0.001). After 1:4 propensity score matching, the association remained significant (26.47% vs. 9.60%; OR = 3.27, 95% CI: 1.45–7.41, *p* = 0.004) (Table 2).

In the matched cohort, the incidence of severe FGR was 13.73% (14/102) in the high-concentration group versus 0.76% (3/396) in the control group (OR = 13.00, 95% CI: 1.70–99.37, *p* = 0.013). The incidence of FGR first detected before 32 weeks was 22.54% (23/102) versus 8.33% (33/396) (OR = 2.75, 95% CI: 1.19–6.39, *p* = 0.018). However, these subgroup analyses were exploratory, and the wide confidence intervals—particularly for severe FGR—reflect limited statistical power; our definition of FGR first detected before 32 weeks—based on the timing of first ultrasound rather than confirmed diagnosis per Delphi criteria and may not be directly comparable with studies using stricter definitions. These findings should be interpreted with caution and require confirmation in larger studies.

In a sensitivity analysis, we additionally adjusted for late-pregnancy maternal and fetal factors—including preeclampsia, gestational diabetes mellitus, and placental abruption—using multivariable conditional logistic regression. The adjusted OR for FGR was 3.29 (95% CI: 1.26–8.58, *p* = 0.015), which remained similar to the primary estimate. This stability supports the robustness of the primary findings under alternative model specifications. These variables were not included in the primary model as they may lie on the causal pathway between high t-cfDNA concentration and FGR.

### 3.3. Secondary Pregnancy Outcomes

In the matched cohort, significant differences were observed between the high-concentration and qualified-concentration groups for secondary pregnancy outcomes. The median gestational age at delivery was significantly lower in the high-concentration group (37.44 weeks, IQR: 37–39) compared with the control group (38.48 weeks, IQR: 38–39) (*p* < 0.001). The rate of preterm birth (<37 weeks) was significantly higher in the high-concentration group (18.63%, 19/102) than in the control group (7.57%, 30/396) (*p* = 0.025) (Table 3).

## 4. Discussion

In this large-scale NIPS-based cohort study, high maternal plasma t-cfDNA concentration (≥ 0.6 ng/µL) in early pregnancy was independently associated with an approximately increased risk of FGR (OR = 3.27, 95% CI: 1.45–7.41), as well as with lower gestational age at delivery and higher preterm birth rates. These findings suggest that a routinely available pre-analytical quality control parameter—already measured in all NIPS workflows—may serve as an early risk indicator for adverse pregnancy outcomes.

The t-cfDNA concentration threshold of 0.05~0.6 ng/µL used in this study originates from our institutional NIPS quality control protocol. This threshold was established to ensure adequate DNA quantity for sequencing; our findings indicate that exceeding this threshold may carry biological and clinical significance beyond its intended analytical purpose.

Our exclusion criteria—removing multiple pregnancies, fetal structural anomalies, severe placental/umbilical cord abnormalities, and intrauterine infection—were applied to minimize confounding from known strong FGR risk factors. These conditions, if retained, would have introduced effect sizes substantially larger than that of t-cfDNA itself, potentially obscuring the independent contribution of the exposure [1]. The subsequent 1:4 propensity score matching further balanced pre-analytical covariates known to influence cfDNA concentration, including maternal age, BMI, gestational age, and comorbidities [21,22,23]. This two-stage design—exclusion of extreme confounders followed by matching of baseline covariates—strengthens the analytical validity of our association estimate.

**Figure 3:**
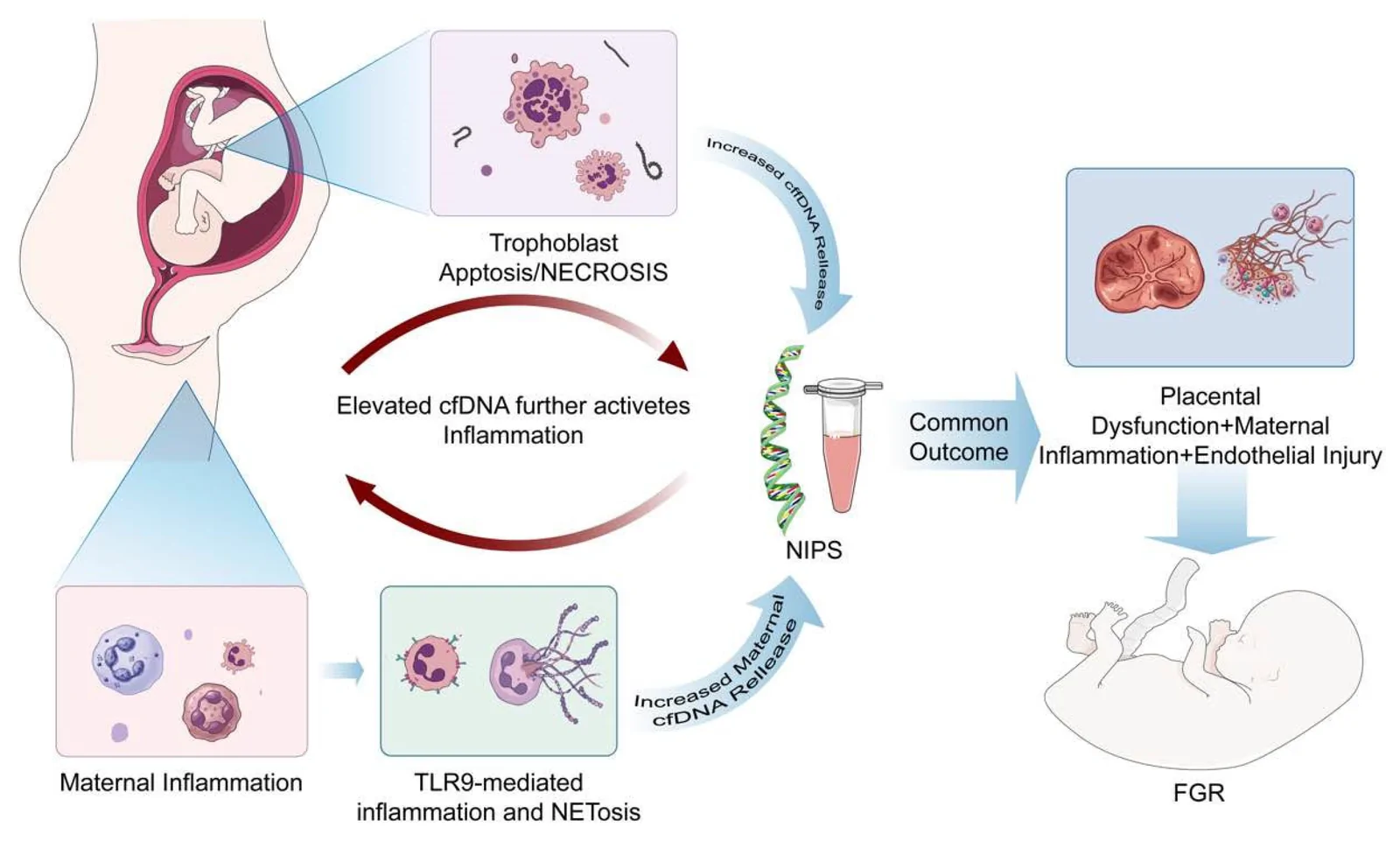
Proposed schematic diagram of the pathophysiological pathways linking high maternal plasma t-cfDNA concentration to FGR. Note: This figure represents a working hypothesis based on the observed associations and published evidence and does not imply direct causal proof from the present study.

T-cfDNA in maternal plasma comprises both fetal-derived (cffDNA) and maternal-derived components. The fetal component originates predominantly from apoptosis and necrosis of placental syncytiotrophoblasts [24,25]. Chronic placental hypoxia can induce excessive trophoblast apoptosis, releasing large amounts of cfDNA into the maternal circulation [26]. Our observation that high t-cfDNA is associated with subsequent FGR is consistent with this mechanistic pathway. Figure 3 presents a proposed schematic diagram integrating the key pathophysiological pathways linking high maternal plasma t-cfDNA concentration to FGR.

Additionally, elevated cfDNA activates Toll-like receptor 9 (TLR9), promoting the release of pro-inflammatory cytokines, including IL-1β and TNF-α [27,28,29]. During neutrophil extracellular trap (NET) formation, released cfDNA and citrullinated histones can damage endothelial cells and placental tissue [30,31]. These pathways converge on impaired placental perfusion, ultimately leading to FGR—the clinical endpoints observed in our high-concentration group.

The schematic also highlights the clinical translation of this pathway: because t-cfDNA is already measured as a pre-sequencing quality control parameter, it can be leveraged as an early risk indicator without additional laboratory cost or workflow modification. This positions t-cfDNA as a readily available biomarker for early risk stratification in NIPS failure cases, with the potential to trigger enhanced ultrasound surveillance and timely intervention.

A cross-sectional study by Rafaeli-Yehudai et al. reported no significant difference in maternal serum t-cfDNA between FGR and normal pregnancies at the time of FGR diagnosis [17]. This discrepancy likely reflects differences in timing (early pregnancy vs. diagnosis) and sample type (plasma vs. serum), suggesting that our study and theirs address distinct temporal windows rather than providing contradictory evidence. Evidence from other pregnancy complications further supports t-cfDNA as a marker of maternal pathology. In preeclampsia—which shares placental dysfunction with FGR—Kolarova et al. found that total cfDNA at diagnosis was more than 10-fold higher than in normal pregnancies, whereas the placental-derived fraction did not differ [11], suggesting that elevated t-cfDNA predominantly reflects maternal tissue injury or systemic inflammation rather than placental shedding alone. This finding also helps explain why low FF—a dilution effect caused by increased maternal-derived cfDNA—is associated with FGR [13,14]. Our previous study showed that high cfDNA is associated with preterm birth [32], consistent with the present findings. A recent PLOS Medicine study by Guo et al. demonstrated that cfDNA profiling can predict preterm birth [33]. However, their approach requires whole-genome sequencing and machine learning, whereas our finding is simpler: A single quality control metric (t-cfDNA concentration) already available in all NIPS laboratories. This lower-tech approach may be more readily implemented in resource-limited settings.

Of note, FF is calculated after sequencing completion, which may introduce reporting delays. In contrast, t-cfDNA concentration—the upstream measure from which FF is derived—is obtained during routine pre-sequencing quality control, potentially offering a time advantage of several days. Therefore, while FF remains a valuable research tool, t-cfDNA concentration may be more amenable to real-time clinical risk assessment.

Approximately 0.2% of pregnant women experience NIPS failure due to high t-cfDNA and lack evidence-based counseling. Our study provides risk information for this population: high t-cfDNA is associated with an approximately increased risk of FGR, as well as lower gestational age and higher preterm birth rates. As a routine quality control parameter, t-cfDNA requires no additional cost, offering health economic advantages. While our findings demonstrate a statistically significant association between high maternal t-cfDNA concentration and FGR, the translation of such associations into clinically useful predictive tools requires careful evaluation. Świercz et al., drawing on a first-trimester screening cohort, conducted a series of studies consistently demonstrating that statistical associations of early pregnancy biomarkers do not equate to clinical predictive utility. Although first-trimester markers—including PAPP-A, fβ-hCG, and ultrasound parameters—showed significant associations with preterm delivery and adverse neonatal outcomes, their predictive performance remained modest [34,35]. These findings underscore a critical principle for the translation of biomarkers: statistical significance alone does not guarantee clinically meaningful predictive accuracy. The same caution should be applied to t-cfDNA, and its clinical utility as a screening biomarker for FGR should be rigorously evaluated in prospective studies—ideally in combination with other established early-pregnancy markers—before it can be recommended for routine practice.

This study has several limitations. First, the retrospective design limits causal inference. Second, the single-center design and the threshold derived from our institutional SOP limit generalizability, the upper concentration limit of 0.6 ng/µL in our center is a technically derived threshold, established based on the official product insert from Berry Genomics and internal statistical quality control data. Third, important potential confounders—including smoking, parity, ART, previous FGR or preeclampsia, and aspirin use—were not available in this retrospective dataset and could not be adjusted for. Fourth, the limited sample size of the high-concentration group (*n* = 102) reduced statistical power for subgroup analyses, as reflected by the wide confidence intervals for severe FGR (OR = 13.00, 95%CI:1.70–99.37) due to only three events in the control group. Our classification of ‘FGR first detected before 32 weeks’ was based on first ultrasound detection of EFW/AC < 10th percentile rather than Delphi criteria, which may limit comparability with studies using stricter definitions. Furthermore, the retrospective design precluded systematic retrieval of ultrasound frequency per participant, potentially introducing detection bias in the timing of first detection. These subgroup findings should therefore be considered exploratory and hypothesis-generating, requiring confirmation in larger studies. Fifth, we acknowledge that our FGR definition based on EFW/AC <10th percentile may include constitutionally small fetuses, as we lacked growth velocity data to distinguish SGA from true pathological FGR per ISUOG/Delphi criteria. This may have diluted the specificity and the observed effect size. Moreover, paternal characteristics—such as height, which has been shown to correlate positively with neonatal birth weight and inversely with SGA risk—were not available in our dataset. As paternal height may influence the baseline fetal growth potential, its absence from our analysis represents an additional source of potential confounding that could have further affected the classification of constitutionally small versus pathologically growth-restricted fetuses [36]. Although PSM balanced known confounders, residual unmeasured confounding cannot be completely excluded. Sixth, although we have now performed conditional logistic regression for preterm birth to account for the matched design, the analysis of gestational age at delivery remains descriptive and did not account for the matched design.

## 5. Conclusions

Maternal plasma t-cfDNA concentration ≥ 0.6 ng/µL in early pregnancy—a routine pre-analytical quality control parameter in NIPS—is significantly associated with an approximately increased risk of FGR and adverse pregnancy outcomes. This readily available, zero-additional-cost metric may hold promise for early risk stratification in women who experience NIPS failure. However, given the observational design and the institution-specific threshold used in this study, its clinical utility requires confirmation in prospective studies with external validation before it can be considered for implementation in routine practice.

## Figures and Tables

**Table 1:** Baseline characteristics of the study population before and after propensity.

Variable	Before PSM						After PSM					
	Total (*n* = 3689)	Qualified-Concentration (*n* = 3581)	High-Concentration (*n* = 108)	Statistic	*p*	SMD	Total (*n* = 498)	Qualified-Concentration (*n* = 396)	High-Concentration (*n* = 102)	Statistic	*p*	SMD
Age, Mean ± SD	30.83 ± 3.26	30.80 ± 3.24	31.70 ± 3.74	t = −2.839	**0.005**	0.241	31.50 ± 3.24	31.47 ± 3.12	31.60 ± 3.67	t = −0.343	0.732	0.034
BMI, M (Q_1_, Q_3_)	21.30 (19.63, 23.34)	21.30 (19.63, 23.34)	21.89 (19.91, 23.61)	Z = −1.365	0.172	0.156	21.50 (19.81, 23.62)	21.48 (19.81, 23.71)	21.66 (19.73, 23.54)	Z = −0.015	0.988	0.018
Gestational age, M (Q_1_, Q_3_)	15.43 (13.43, 16.43)	15.43 (13.43, 16.43)	15.43 (14.43, 17.43)	Z = −1.653	0.098	0.207	15.43 (13.43, 16.43)	15.43 (13.43, 16.43)	15.43 (14.43, 16.43)	Z = −0.140	0.889	0.082
Maternal comorbidities, *n* (%)	31 (0.84)	24 (0.67)	7 (6.48)	−	**<0.001**	0.236	15 (3.01)	11 (2.78)	4 (3.92)	χ^2^ = 0.077	0.781	0.059
Fetal factors, *n* (%)	122 (3.31)	120 (3.35)	2 (1.85)	χ^2^ = 0.343	0.558	−0.111	9 (1.81)	7 (1.77)	2 (1.96)	χ^2^ = 0.000	1	0.014
Placenta factors, *n* (%)	33 (0.89)	32 (0.89)	1 (0.93)	−	1	0.003	2 (0.4)	1 (0.25)	1 (0.98)	−	0.368	0.074

Abbreviations: SD, standard deviation; IQR, interquartile range; SMD, standardized mean difference; BMI, body mass index; NIPS, noninvasive prenatal screening. Detailed definitions of all covariates are provided in Supplementary Table S1.

**Table 2:** Association between high t-cfDNA concentration and FGR.

Outcome	Qualified-Concentration	High-Concentration	OR (95% CI)	*p*
Before matching	*n* = 3581	*n* = 108		
FGR, *n* (%)	328 (9.16)	29 (26.85)	3.64 (2.34~5.66)	<0.001 *
Severe FGR, *n* (%)	35 (0.98)	15 (13.89)	17.65 (9.26~33.63)	<0.001 *
FGR first detected before 32 weeks, *n* (%)	286 (7.99)	24 (22.22)	3.50 (2.18~5.62)	<0.001 *
After matching	*n* = 396	*n* = 102		
FGR, *n* (%)	38 (9.60)	27 (26.47)	3.27 (1.45–7.41)	0.004 *
Severe FGR, *n* (%)	3 (0.76)	14 (13.73)	13.00 (1.70–99.37) ^†^	0.013 *
FGR first detected before 32 weeks, *n* (%)	33 (8.33)	23 (22.54)	2.75 (1.19–6.39) ^†^	0.018 *

Abbreviations: OR, odds ratio; CI, confidence interval; FGR, fetal growth restriction. Note: * *p* < 0.05 was considered statistically significant; OR from conditional logistic regression (primary analysis). ^†^ Exploratory subgroup analyses. These estimates should be interpreted as hypothesis-generating, due to limited statistical power.

**Table 3:** Secondary pregnancy outcomes in the matched cohort.

Outcome	Qualified-Concentration (*n* = 396)	High-Concentration (*n* = 102)	OR (95% CI)	*p*
Gestational age at delivery (weeks), Median (IQR)	38.48 (38–39)	37.44 (37–39)	—	<0.001 *
Preterm birth (<37 weeks), *n* (%)	30 (7.57%)	19 (18.63%)	3.04 (1.15–8.01)	0.025 *

Note: * *p* < 0.05 was considered statistically significant; OR from conditional logistic regression.

## Data Availability

The data for this article are not publicly available because of privacy concerns. Requests to access these datasets should be directed to LHQ, hongqian.liu@163.com.

## Supplementary Materials

The following supporting information can be downloaded at: https://www.mdpi.com/article/10.3390/jcm15176798/s1, Table S1. Definitions and Coding of covariates used in propensity score matching.

## Abbreviations

The following abbreviations are used in this manuscript:

AC	Abdominal Circumference
ACOG	American College of Obstetricians and Gynecologists
BMI	Body Mass Index
t-cfDNA	Total Cell-Free DNA
cffDNA	Cell-Free Fetal DNA
CI	Confidence Interval
EFW	Estimated Fetal Weight
FF	Fetal Fraction
FGR	Fetal Growth Restriction
GDM	Gestational Diabetes Mellitus
IQR	Interquartile Range
NET	Neutrophil Extracellular Trap
NIPS	Noninvasive Prenatal Screening
OR	Odds Ratio
PSM	Propensity Score Matching
RR	Relative Risk
SD	Standard Deviation
SMD	Standardized Mean Difference
SOP	Standard Operating Procedure
TLR9	Toll-like Receptor 9
