# Supplementary material for: Excessively High Maternal Total Cell-Free DNA Concentration and Fetal Growth Restriction in Singleton Pregnancies: A Retrospective Cohort Study
Source: J Clin Med. 2026 Sep 2;15(17):6798. doi: 10.3390/jcm15176798 (PMC13566906; doi:10.3390/jcm15176798)
Supplement: Supplementary file 1 [file jcm-15-06798-s001.zip › jcm-4456439-supplementary.pdf]

Table S1. Definitions and Coding of covariates used in propensity score matching

**Category**	**Specific conditions / definitions**
Maternal age	Age of the pregnant woman at the time of the NIPS blood draw (years), treated as a continuous variable.
BMI	Body mass index calculated from weight and height (kg/m^2), treated as a continuous variable.
Gestational age at NIPS	Gestational age (weeks) at the time of the NIPS blood draw, determined based on the last menstrual period or the first-trimester ultrasound, treated as a continuous variable.
Maternal comorbidities	Presence of any of the following conditions diagnosed before or during early pregnancy (prior to NIPS blood draw) : pregestational diabetes mellitus (type 1 or type 2); gestational diabetes diagnosed in early pregnancy; autoimmune disorders (e.g., systemic lupus erythematosus, antiphospholipid syndrome, rheumatoid arthritis); chronic hypertension (diagnosed before pregnancy or before 20 weeks of gestation); thyroid disease (including hypothyroidism, hyperthyroidism, and Hashimoto's thyroiditis). Coded as 1 if any condition present, 0 otherwise.
Fetal factors	Presence of any ultrasound soft marker detected prior to or at the time of NIPS blood draw that may influence fetal growth assessment or cfDNA concentration, including: echogenic intracardiac focus; choroid plexus cysts; echogenic bowel; mild ventriculomegaly; other transient sonographic findings. Coded as 1 if any marker present, 0 otherwise.
Placental factors	Presence of any of the following placental conditions diagnosed prior to or at the time of NIPS blood draw: asymptomatic placenta previa (without bleeding), marginal cord insertion, and other morphological anomalies of the placenta. These are milder anatomical variants that do not constitute strong independent FGR risk factors. Coded as 1 if any condition present, 0 otherwise.
